# Genetic polymorphisms of long non-coding RNA GAS5 predict platinum-based concurrent chemoradiotherapy response in nasopharyngeal carcinoma patients

**DOI:** 10.18632/oncotarget.19725

**Published:** 2017-07-31

**Authors:** Zhen Guo, Youhong Wang, Yu Zhao, Yi Jin, Liang An, Bin Wu, Zhaoqian Liu, Xiaoping Chen, Honghao Zhou, Hui Wang, Wei Zhang

**Affiliations:** ^1^ Department of Clinical Pharmacology, Xiangya Hospital, Central South University and Institute of Clinical Pharmacology, Central South University, Hunan Key Laboratory of Pharmacogenetics, Changsha, 410008, P.R. China; ^2^ Key Laboratory of Translational Radiation Oncology, Hunan Province, Department of Radiation Oncology, Hunan Cancer Hospital and The Affiliated Cancer Hospital of Xiangya School of Medicine, Central South University, Changsha, 410013, P.R. China

**Keywords:** nasopharyngeal carcinoma, chemoradiotherapy, toxicity, GAS5, polymorphisms

## Abstract

LncRNA *GAS5* plays a tumor suppressive role in a variety of human cancers and promises to be a novel diagnostic biomarker, therapy target, as well as prognostic biomarker. However, the role of *GAS5* in nasopharyngeal carcinoma (NPC) remains elusive. The objective of the present study was to evaluate the effect of single nucleotide polymorphisms (SNPs) in *GAS5* on treatment efficacy and toxicity in NPC patients receiving chemoradiotherapy. Three potentially functional SNPs of *GAS5* were genotyped in 267 NPC patients and validated in another 238 NPC patients treated with chemoradiotherapy from southern China. Multivariate logistic regression analyses and stratification analyses were used to estimate the association of candidate SNPs and chemoradiotherapy efficacy and toxic reactions. Our results showed that rs2067079 kept a consistent association with severe myelosuppression and severe neutropenia in discovery set (OR=2.403, P=0.009; OR=2.454, P=0.015; respectively), validation set (OR=3.653, P=0.027; OR=4.767, P=0.016; respectively), and combined dataset (OR=1.880, P=0.007; OR=2.079, P=0.005; respectively). rs2067079 CT genotype carriers presented an even more remarkable increased risk of severe myelosuppression (OR=3.878, P=0.003) and severe neutropenia (OR=3.794, P=0.009) in subgroups taking paclitaxel+platinum as concurrent chemoradiotherapy regimen. Besides, we found a gene-does effect of rs6790, with the incidence rate of severe myelosuppression decreased from 23.56% to 17.21% to 10% and the incidence rate of severe neutropenia decreased from 30.4% to 20.9% to 17.1% for rs6790 GG *vs* GA *vs* AA genotype carriers. Our results indicate the potential role of lncRNA GAS5 polymorphisms rs2067079 and rs6790 as predictive biomarkers for chemoradiotherapy induced toxic reactions in NPC patients.

## INTRODUCTION

Nasopharyngeal carcinoma (NPC) is one of the most common malignant tumors in Southern China and Southeast Asia, with the incidence as high as 20∼50 of 100,000, which is 20∼50 times higher of Western world (1 of 100,000) [1]. Currently, radiation therapy is a key modality in the treatment of NPC. Moreover, when radiotherapy is combined with chemotherapy or other targeted therapies, treatment efficiency is improved [2]. However, there still have a small group of patients have to suffer chemoradiotherapy resistance and a spectrum of severe side effects (toxicities) [3]. The occurrence of serious toxicity and treatment resistance not only bring pain for the patients but also block the scheduled treatment procedure. It is not unusual to see that a large patient-to-patient variability exists in chemoradiotherapy response despite uniform treatment protocols. Although some of this may be ascribed to comorbidities, body habitus and stochastic factors, it has become increasingly believed that genetic background takes a leading role in the interindividual variation in chemoradiotherapy response [4].

Long non-coding RNAs (lncRNAs) are noncoding RNAs with more than 200 nucleotide in length. Although with limited protein coding capability, lncRNAs play an important role in a number of biological processes, including chromatin modification, transcription processing, RNA-RNA interactions, and post-transcription regulation [5]. Increasing evidence have indicated lncRNA played a role in carcinogenesis and could be diagnostic or prognostic biomarkers for cancer. In the past decades, numerous studies have linked genetic variation in miRNA (a kind of non-coding RNA) sequences to cancer risk and prognosis, yet little is known about lncRNA SNPs [6]. Genetic variants in lncRNAs have the potential to exert broad impact as they can affect their biogenesis, processing, and target site binding in a variety of ways.

Located at 1q25, growth arrest-specific 5 (*GAS5*) encodes multiple small nucleolar RNAs (snoRNAs) within its introns, while exonic sequences produce lncRNA which can act as a riborepressor of the glucocorticoid and related receptors [7]. lncRNA *GAS5* was previously identified to be down-regulated and functions as a tumor suppressor gene in many kinds of cancers, including breast cancer, prostate cancer, pancreatic cancer, bladder cancer, lung cancer, gastric cancer, glioma, hepatocellular carcinoma, cervical cancer, pleural mesothelioma and so on [8-17]. *GAS5* plays a pivotal role in the control of cell survival and proliferation by sensitizing mammalian cells to apoptosis and promoting G1 cell cycle arrest [18]. Accumulating studies have identified *GAS5* as a potential biomarker for cancer susceptibility, metastasis, as well as survival. Nevertheless, no one has linked *GAS5* SNP with NPC treatment responses [15, 16, 19-21].

In current study, we aim to evaluate the effects of SNPs in lncRNA *GAS5* on clinical early toxic reactions and treatment efficacy in patients with nasopharyngeal carcinoma receiving chemoradiotherapy in a Chinese population. It is of importance to increase our understanding of the molecular pathogenesis of chemoradiotherapy response, find ways of predicting those patients likely to suffer with long term side effects and treatment resistance and develop new approaches for their amelioration.

## RESULTS

### Subjects characteristics and genotyping

The patient characteristics in the discovery stage and the validation stage were listed in Table 1. The median age at the time of diagnosis was 47 years (ranging 15–73 years) for all the 505 patients. There were 374 male and 131 female patients, with a male-to-female ratio of 2.85. Most of the patients (90.1%) were diagnosed at late stages (III and IV), and the remaining (9.9%) were at early stages (I and II). All the patients received IMRT radiation technique and treated with IC plus CCRT regimen. The average pGTVnx irradiation dose was 71.34 Gy with NDP as the mostly used concurrent chemotherapy regimen. The toxic reactions incidence rate was 10.1% for severe dermatitis, 25.5% for severe oral mucositis, 24.0% for severe myelosuppression, 18.6% for severe neutropenia, 14.5% for severe leukopenia, 43.0% for anemia, and 19.4% for thrombocytopenia during the CCRT treatment period.

**Table 1:** Patient demographics and clinical characteristics

Patient characteristics	Discovery stage (N=267)	Validation stage (N=238)	Combined cohort (N=505)
Gender			
Male	205 (76.78)	169 (71.0)	374 (74.1)
Female	62 (23.22)	69 (29.0)	131 (25.9)
Age, years			
Mean±SD	47.14±9.50	47.71±8.75	47.41±9.15
< 47	128 (47.94)	101 (42.4)	229 (45.3)
≥ 47	139 (52.06)	137 (57.6)	276 (54.7)
BMI			
< 18.5	17 (6.37)	13 (5.5)	30 (5.9)
18.5 ∼ 24	148 (55.43)	126 (52.9)	274 (54.3)
≥ 24	102 (38.2)	99 (41.6)	201 (39.8)
Smoking status			
Smoker	129 (48.31)	118 (49.6)	247 (48.9)
Nonsmoker	138 (51.69)	120 (50.4)	258 (51.1)
Drinking status			
Drinker	47 (17.60)	43 (18.9)	90 (17.8)
Nondrinker	220 (82.40)	195 (81.9)	415 (82.2)
Histological type			
WHO type II	114 (42.70)	100 (42.0)	214 (42.4)
WHO type III	153 (57.30)	138 (58.0)	291 (57.6)
Clinical stage^a^			
I-II	28 (10.49)	22 (9.2)	50 (9.9)
III-IV	239 (89.51)	216 (90.8)	455 (90.1)
T-staging			
T1-T2	139 (50.06)	107 (45.0)	246 (48.7)
T3-T4	128 (49.94)	131 (55.0)	259 (51.3)
N-staging			
N0-N1	52 (19.47)	41 (17.2)	93 (18.4)
N2-N3	215 (80.53)	197 (82.8)	412 (81.6)
IC regimen			
DP	94 (35.20)	106 (44.5)	200 (39.6)
FP	54 (20.23)	38 (16.0)	92 (18.2)
TP	109 (40.82)	94 (39.5)	203 (40.2)
GP	10 (3.75)	0 (0)	10 (2)
CCRT regimen			
FP	49 (18.35)	36 (15.1)	85 (16.8)
TP	51 (19.10)	57 (23.9)	108 (21.4)
DDP	35 (13.11)	48 (20.2)	83 (16.4)
NDP	108 (40.45)	64 (26.9)	172 (34.1)
DP	24 (8.99)	33 (13.9)	57 (11.3)
pGTVnx (irradiation dose)			
Mean±SD	71.28±1.89	71.42±3.537	71.34±2.79
< 71.00Gy	153 (57.30)	100 (42.0)	261 (51.7)
≥71.00Gy	114 (42.7)	138 (58.0)	234 (48.3)
CCRT- induced toxic reactions^b^			
Grade 3-4 Dermatitis	22 (8.2)	29 (12.2)	51 (10.1)
Grade 3-4 Oral mucositis	57 (21.3)	72 (30.3)	129 (25.5)
Grade 3-4 Myelosuppression	60 (22.5)	61 (25.6)	121 (24.0)
Grade 3-4 Neutropenia	50 (18.7)	44 (18.5)	94 (18.6)
Grade 3-4 Leukopenia	36 (13.5)	37 (15.5)	73 (14.5)
Anemia	105 (39.3)	112 (47.1)	217 (43.0)
Thrombocytopenia	56 (21.0)	42 (17.6)	98 (19.4)

BMI, Body mass index.

CCRT, Concurrent chemoradiotherapy.

IC, Induction chemotherapy.

DP, Docetaxel + Cisplatin/Nedaplatin.

FP, 5-fluorouracil + Cisplatin/Nedaplatin.

TP, Paclitaxel + Cisplatin/Nedaplatin.

GP, Gemcitabine + Cisplatin/Nedaplatin.

DDP, Cisplatin alone.

NDP, Nedaplatin alone.

^a^2002 American Joint Committee on Cancer (AJCC) staging system.

^b^Common Terminology Criteria for Adverse Events (CTCAE 3.0).

All of the three SNPs in both discovery set and validation set showed a call rate > 99%, and the genotype distributions were in accordance with HWE (p>0.05). Besides, the allele frequency in our study cohort was similar to the MAF of 1000 Genomes CHS database, as shown in Table 2.

**Table 2:** Characteristics of the candidate SNPs

SNP	Chromosome	Position	Localization	Regulatory feature	miRNA-lncRNA target gain or loss	Call rate (Discovery Stage)	Call rate (Validation Stage)	MAF (CHS)	Allele frequency	HWE (Discovery Stage/Validation Stage/Combined Stage)
rs2067079	1	173866073	Intron	Promoter/Enhancer	√	100%	99.2%	0.271	0.272	0.405/0.549/0.292
rs6790	1	173865494	Exon	Promoter/Enhancer	×	100%	100%	0.395	0.380	0.173/0.709/0.571
rs17359906	1	173867056	Intron	Promoter/Enhancer	√	100%	---	0.081	0.054	0.800/--/--

HWE, Hardy-Weinberg equilibrium; MAF, minor allele frequency.

### *GAS5* SNPs and CCRT induced hematotoxicities

Multivariate logistic regression analysis was performed to identify the role of rs2067079, rs6790, rs17359906 on CCRT induced hematotoxicities in the discovery set of 267 NPC patients. We found that rs2067679 was significantly associated with severe myelosuppression (CT *vs* CC, OR=2.403, P=0.009), severe neutropenia (CT *vs* CC, OR=2.454, P=0.015), and severe leukopenia (CT *vs* CC, OR=2.938, P=0.011), but not anemia and thrombocytopenia (Table 3, Table 4, Supplementary Table 1, Supplementary Table 2). rs6790 displayed a decreased risk of anemia with an OR of 0.501 (GA *vs* GG, P=0.041) (Supplementary Table 2). While no significant association was found between rs17359906 and any hematotoxicities, so we didn’t genotype it in the following validation stage.

**Table 3:** Multivariate logistic regression analysis of candidate SNPs and their association with concurrent chemoradiotherapy induced grade >2 myelosuppression in NPC patients

Genotypes	Discovery Stage				Validation Stage				Combined Stage			
	Myelosuppression		OR^a^ (95% CI)	P ^a^	Myelosuppression		OR^a^ (95% CI)	P ^a^	Myelosuppression		OR^a^ (95% CI)	P ^a^
	Grade ≤2 N (%)	Grade >2 N (%)			Grade ≤2 N (%)	Grade >2 N (%)			Grade ≤2 N (%)	Grade >2 N (%)		
rs2067079												
CC	115(55.6)	23(38.3)	1.00 (reference)		106 (59.9)	27 (44.3)	1.00 (reference)		221 (57.6)	50 (41.3)	1.00 (reference)	
CT	70(33.8)	34(56.7)	2.403 (1.240-4.658)	**0.009**	60 (33.9)	26 (42.6)	1.566 (0.811-3.024)	0.181	130 (33.9)	60 (49.6)	1.880 (1.184-2.984)	**0.007**
TT	22(10.6)	3(5.0)	0.595 (0.142-2.484)	0.476	9 (5.1)	8 (13.1)	3.653 (1.162-11.478)	**0.027**	31 (8.1)	11 (9.1)	1.508 (0.663-3.430)	0.327
TT+CT vs CC			1.988 (1.048-3.772)	**0.035**			3.258 (1.079-9.843)	**0.036**			1.813 (1.164-2.824)	**0.009**
TT vs CT+CC			0.385 (0.093-1.592)	0.187			1.807 (0.970-3.367)	0.062			1.169 (0.525-2.602)	0.702
rs6790												
GG	83 (40.1)	26 (43.3)	1.00 (reference)		50 (28.2)	32 (52.5)	1.00 (reference)		133 (34.6)	58 (47.9)	1.00 (reference)	
GA	101 (48.8)	30 (50.0)	0.794 (0.402-1.568)	0.507	92 (52.0)	21 (34.4)	0.341 (0.170-0.684)	**0.002**	193 (50.3)	51 (42.1)	0.538 (0.336-0.861)	**0.010**
AA	23 (11.1)	4 (6.7)	0.636 (0.182-2.229)	0.480	35 (19.8)	8 (13.1)	0.360 (0.140-0.923)	**0.033**	58 (15.1)	12 (9.9)	0.454 (0.218-0.946)	**0.035**
AA+GA vs GG			0.767 (0.399-1.476)	0.428			0.591 (0.241-1.451)	0.251			0.519 (0.332-0.811)	**0.004**
AA vs GA+GG			0.716 (0.214-2.392)	0.587			0.317 (0.161-0.626)	**0.001**			0.628 (0.312-1.264)	0.193
rs17359906												
GG	188 (90.8)	51 (85.0)	1.00 (reference)		--	--	--		--	--	--	
GA	18 (8.7)	9 (15.0)	1.784 (0.645-4.936)	0.265	--	--	--	--	--	--	--	--
AA	1 (0.5)	0 (0)	--	--	--	--	--	--	--	--	--	--
AA+GA vs GG			1.758 (0.637-4.853)	0.276			--	--			--	--
AA vs GA+GG			--	--			--	--			--	--

^a^ Adjusted for gender, age, BMI, smoking status, drinking status, histological type, clinical stage, IC regimen, CCRT regimen, and pGTVnx irradiation dose.

P < 0.05 was shown in bold.

**Table 4:** Multivariate logistic regression analysis of candidate SNPs and their association with concurrent chemoradiotherapy induced grade >2 neutropenia in NPC patients

Genotypes	Discovery Stage				Validation Stage				Combined Stage			
	Neutropenia		OR^a^ (95% CI)	P ^a^	Neutropenia		OR^a^ (95% CI)	P ^a^	Neutropenia		OR^a^ (95% CI)	P ^a^
	Grade ≤2 N (%)	Grade >2 N (%)			Grade ≤2 N (%)	Grade >2 N (%)			Grade ≤2 N (%)	Grade >2 N (%)		
rs2067079												
CC	120 (55.3)	18 (36.0)	1.00 (reference)		115 (59.3)	18 (40.9)	1.00 (reference)		235 (57.2)	36 (38.3)	1.00 (reference)	
CT	75 (34.6)	29 (58.0)	2.454 (1.190-5.061)	**0.015**	66 (34.0)	20 (45.5)	1.823 (0.851-3.906)	0.123	141 (34.3)	49 (52.1)	2.079 (1.245-3.470)	**0.005**
TT	22 (10.1)	3 (6.0)	0.730 (0.166-3.210)	0.677	11 (5.7)	6 (13.6)	4.767 (1.334-17.036)	**0.016**	33 (8.0)	9 (9.6)	1.665 (0.682-4.065)	0.263
TT+CT vs CC			2.067 (1.027-4.161)	**0.042**			3.907 (1.148-13.295)	**0.029**			2.004 (1.225-3.278)	**0.006**
TT vs CT+CC			0.519 (0.122-2.216)	0.376			2.134 (1.037-4.392)	**0.040**			1.211 (0.512-2.862)	0.663
rs6790												
GG	87 (40.1)	22 (44.0)	1.00 (reference)		59 (30.4)	23 (52.3)	1.00 (reference)		146 (35.5)	45 (47.9)	1.00 (reference)	
GA	106 (48.8)	25 (50.0)	0.828 (0.396-1.730)	0.615	96 (49.5)	17 (38.6)	0.460 (0.214-0.990)	**0.047**	202 (49.1)	42 (44.7)	0.585 (0.348-0.984)	**0.043**
AA	24 (11.1)	3 (6.0)	0.615 (0.149-2.532)	0.500	39 (20.1)	4 (9.1)	0.258 (0.076-0.872)	**0.029**	63 (15.3)	7 (7.4)	0.326 (0.132-0.805)	**0.015**
AA+GA vs GG			0.793 (0.389-1.617)	0.524			0.362 (0.113-1.158)	0.087			0.525 (0.319-0.865)	**0.011**
AA vs GA+GG			0.679 (0.174-2.654)	0.578			0.402 (0.195-0.830)	**0.014**			0.439 (0.186-1.037)	0.060
rs17359906												
GG	198 (91.2)	41 (82.0)	1.00 (reference)		--	--	--		--	--	--	
GA	18 (8.3)	9 (18.0)	2.255 (0.791-6.430)	0.128	--	--	--	--	--	--	--	--
AA	1 (0.5)	0 (0)	--	--	--	--	--	--	--	--	--	--
AA+GA vs GG			2.119 (0.797-5.636)	0.132			--	--			--	--
AA vs GA+GG			--	--			--	--			--	--

^a^ Adjusted for gender, age, BMI, smoking status, drinking status, histological type, clinical stage, IC regimen, CCRT regimen, and pGTVnx irradiation dose.

P < 0.05 was shown in bold.

The promising rs2067079 and rs6790 were further genotyped in 238 additional NPC patients in the validation stage. Our result revealed that only rs2067679 showed consistently significant association with severe myelosuppression (TT *vs* CC, OR=3.653, P=0.027) and severe neutropenia (TT *vs* CC, OR=4.767, P=0.016) in the validation stage (Table 3, Table 4). Though rs6790 didn’t show any association with severe myelosuppression and severe neutropenia in the discovery stage, rs6790 demonstrated a decreased risk of severe myelosuppression (GA *vs* GG, OR=0.341, P=0.002) and severe neutropenia (AA *vs* GG, OR=0.258, P=0.029) in the validation stage (Table 3, Table 4).

**Figure 1:**
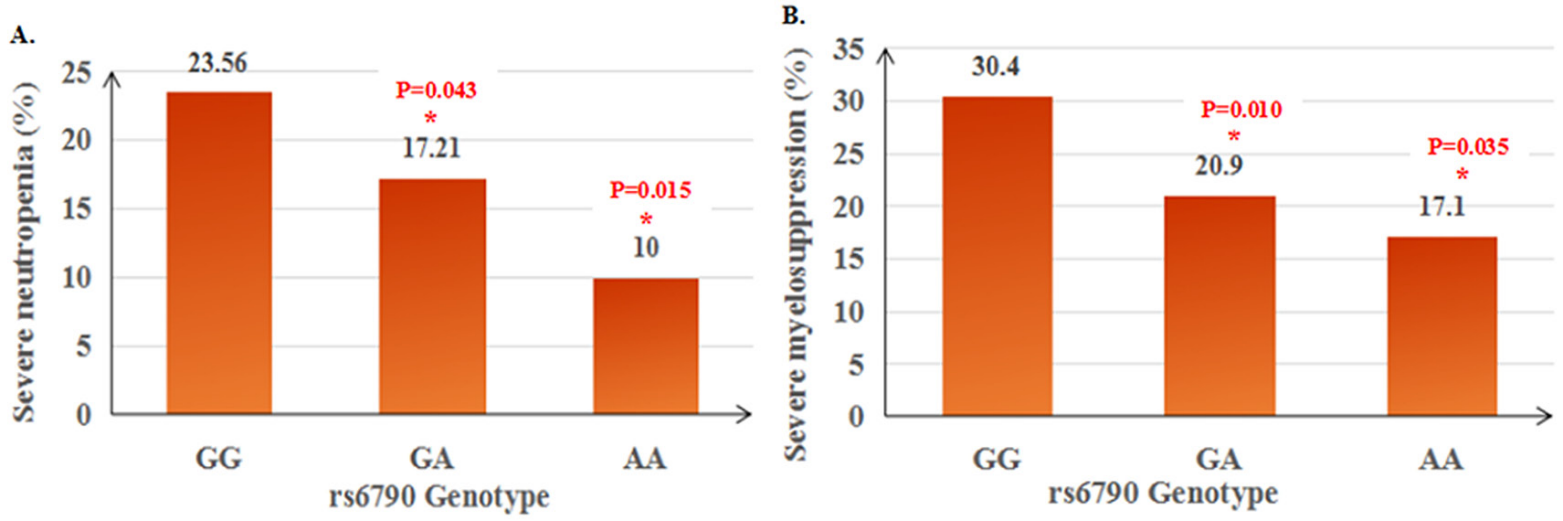
Gene-dose effect of rs6790 on concurrent chemoradiotherapy induced severe neutropenia **(A)** and severe myelosupression **(B)** in NPC patients.

Moreover, combined analysis showed that rs2067079 was still significantly associated with severe myelosuppression (CT *vs* CC, OR=1.880, P=0.007), severe neutropenia (CT *vs* CC, OR=2.079, P=0.005), and severe leukopenia (CT *vs* CC, OR=1.901, P=0.023) (Table 3, Table 4, Supplementary Table 1). Besides, rs6790 displayed a similar trend to the validation stage, which was linked to a decreased risk of severe myelosuppression (GA *vs* GG, OR=0.538, P=0.010) and severe neutropenia (AA *vs* GG, OR=0.326, P=0.015) in the combined cohort (Table 3, Table 4). Noteworthy, there exist a gene-does effect of rs6790, with the incidence rate of severe myelosuppression decreased from 23.56% to 17.21% to 10% for rs6790 GG *vs* GA *vs* AA genotype carriers. Likely, the incidence rate of severe neutropenia decreased from 30.4% to 20.9% to 17.1% for rs6790 GG *vs* GA *vs* AA genotype carriers (Figure 1).

**Figure 2:**
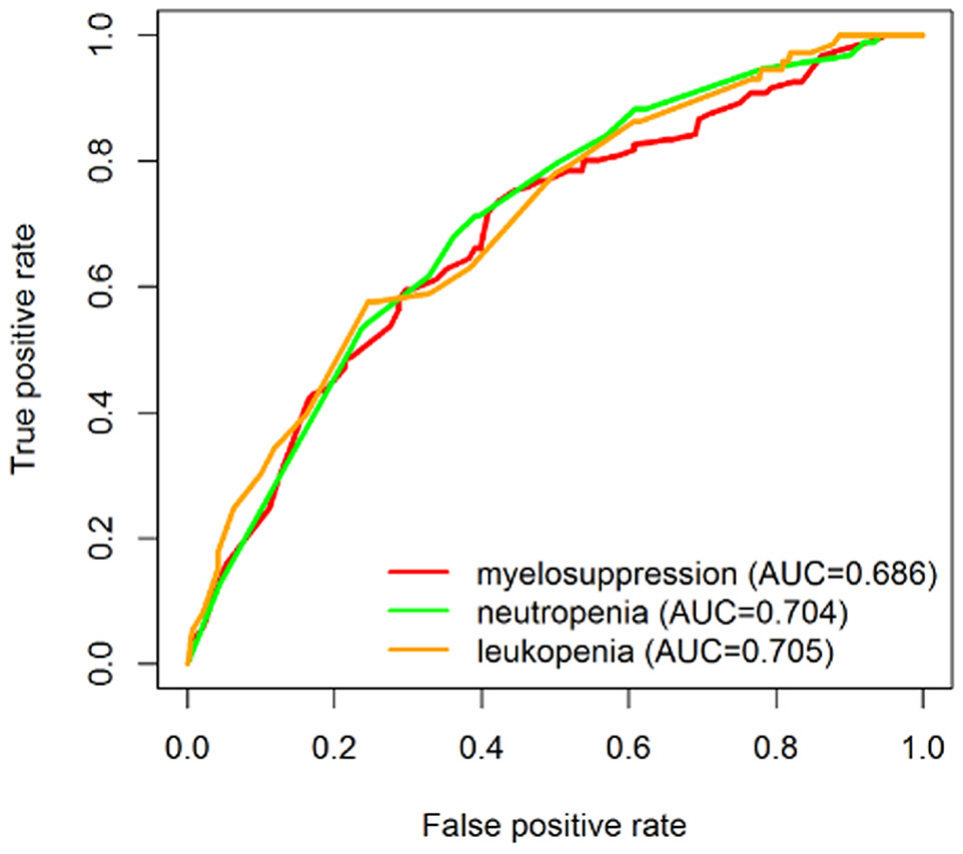
ROC curves showing the discriminatory power to predict chemoradiotherapy induced severe toxicities

To further obtain the predictive power of rs2067079 and rs6790, a risk score model was build to predict each patient’s risk of developing hematotoxicities by logistic regression based on the two SNPs and clinical features. The receiver operating characteristic curve (ROC) was used to compute the sensitivity and specificity of toxicity prediction. The area under the curve (AUC) of the risk score model were 0.686, 0.704, 0.705 for severe myelosuppression, neutropenia, and leukopenia, respectively, indicating a moderate performance (Figure 2).

### *GAS5* SNPs and CCRT induced severe dermatitis and oral mucotisis

As for CCRT induced severe dermatitis and oral mucotisis with NPC patients, we only found a slight relationship in the discovery stage for severe oral mucotisis and rs2067079 (CT *vs* CC, OR=1.928, P=0.049), as well as rs6790 (GA *vs* GG, OR=0.511, P=0.049). Neither of the two SNPs were verified in the validation stage, nor in the combined cohort (Supplementary Table 3). Nevertheless, no significant evidence was found for interaction between these candidate SNPs and severe dermatitis in NPC patients receiving CCRT treatment.

### Stratification analyses of *GAS5* rs2067079, rs6790 and CCRT induced early toxic reactions

Furthermore, we evaluated the stratified association of *GAS5* rs2067079 and rs6790 with CCRT induced early toxicities by irradiation dose and chemotherapy regimens, since these factors were disequilibrium in severe and mild toxicity groups. We observed that the rs2067079 CT genotype carriers emerged an even more remarkable increased risk of severe myelosuppression (CT *vs* CC, OR=3.878, P=0.003) in subgroups of CCRT regimen-TP. Moreover, rs2067079 also presented a dramatic increased effect on severe neutropenia in subgroups of CCRT regimen-TP (CT *vs* CC, OR=3.794, P=0.009) and subgroups of CCRT regimen-NDP (TT *vs* CC, OR=5.085, P=0.028). Though rs2067079 may act as a risk factor for severe myelosuppression and neutropenia, it seems that rs2067079 played a favorable role for CCRT induced thrombocytopenia in subgroups taking TP as IC regimen (CT *vs* CC, OR=0.394, P=0.034) and DDP as CCRT regimen (CT *vs* CC, OR=0.101, P=0.047). Besides, patients of rs2067079 TT genotype receiving DP for IC regimen (TT *vs* CC, OR=3.031, P=0.047) or CCRT regimen (TT *vs* CC, OR=21.882, P=0.043) were subjected to high risk of oral mucotisis (Table 5).

**Table 5:** Stratified analyses of GAS5 rs2067079, rs6790 and CCRT induced early toxicities among NPC patients by irradiation dose and chemotherapy regimens

Subgroups	SNP	Toxic reaction	Grade ≤2 WW/WM/MM	Grade >2 WW/WM/MM	OR^a^ (95% CI)	P ^a^
IC regimen- TP	rs2067079	Myelosuppression	72/50/13	25/34/9	2.010 (1.045-3.867)	**0.037**
		Neutropenia	76/55/15	21/29/7	2.093 (1.026-4.268)	**0.042**
		Thrombocytopenia*	74/73/21	23/11/1	0.394 (0.166-0.931)	**0.034**
IC regimen- DP	rs2067079	Oral mucotisis	80/50/8	27/25/8	3.031 (1.014-9.055)	**0.047**
CCRT regimen- TP	rs2067079	Myelosuppression	34/21/5	15/28/5	3.878 (1.562-9.626)	**0.003**
		Neutropenia	36/25/7	13/24/3	3.794 (1.403-10.260)	**0.009**
CCRT regimen- DP	rs2067079	Oral mucotisis	24/21/1	4/5/2	21.882 (1.095-437.387)	**0.043**
	rs6790	Myelosuppression	10/24/7	9/5/2	0.008 (0.000-0.179)	**0.002**
		Neutropenia	11/25/8	8/4/1	0.026 (0.002-0.325)	**0.005**
		Leukopenia	14/25/7	5/4/2	0.113 (0.014-0.898)	**0.039**
CCRT regimen- NDP	rs2067079	Neutropenia	81/56/14	7/9/4	5.085 (1.189-21.746)	**0.028**
	rs6790	Anemia*	43/65/12	25/21/6	0.405 (0.182-0.898)	**0.026**
CCRT regimen- DDP	rs2067079	Thrombocytopenia*	37/23/7	12/2/1	0.101 (0.011-0.966)	**0.047**
CCRT regimen- FP	rs6790	Dermatitis	24/32/17	7/2/3	0.026 (0.001-0.464)	**0.013**
pGTVnx < 71.00Gy	rs2067079	Myelosuppression	115/76/18	20/25/6	2.059 (1.020-4.158)	**0.044**
		Neutropenia	123/81/20	12/20/4	2.859 (1.254-6.517)	**0.012**
		Leukopenia	123/83/20	12/18/4	2.533 (1.078-5.951)	**0.033**
	rs6790	Myelosuppression	75/107/28	26/21/4	0.486 (0.241-0.980)	**0.044**
		Neutropenia	82/113/30	19/15/2	0.439 (0.198-0.973)	**0.043**
		Anemia*	63/89/15	38/39/17	0.493 (0.261-0.933)	**0.030**
pGTVnx ≥71.00Gy	rs2067079	Myelosuppression	106/54/13	30/35/5	2.387 (1.235-4.614)	**0.010**
		Neutropenia	112/60/13	24/29/5	2.273 (1.128-4.580)	**0.022**

*NOTE: the two groups for anemia and thrombocytopenia was assigned as grade=0 and grade >0 as the number of patients developing grade >2 anemia and thrombocytopenia were too little.

WW: wild homozygote; WM: heterozygote; MM: mutant homozygote.

^a^ adjusted for the remaining clinical covariates except for the stratified factor.

P < 0.05 was shown in bold.

Notably, rs6790 displayed a prominently decreased risk for severe myelosuppression (GA *vs* GG, OR=0.008, P=0.002), severe neutropenia (GA *vs* GG, OR=0.026, P=0.005), and severe leukopenia (GA *vs* GG, OR=0.113, P=0.039) in subgroups taking DP as the CCRT regimen. We also observed that the risk of anemia decreased to 0.405 and 0.493 in subgroups of CCRT regimen-NDP (GA *vs* GG, P=0.026) and pGTVnx < 71.00Gy (GA *vs* GG, P=0.030), respectively. Besides, rs6790 GA genotype carriers were not likely to experience severe dermatitis in subgroups receiving FP for CCRT regimen (GA *vs* GG, OR=0.026, P=0.013) (Table 5).

### *GAS5* SNPs and chemoradiotherapy efficacy

414 patients with 3 months MRI follow-up information were involved in the treatment efficacy analysis. The effective remission (PR+CR) rate was 93.96% for nasopharyngeal primary tumor and 85.02% for neck lymph nodes. Multivariate logistic regression results showed that none of the three SNPs were associated with either primary tumor treatment efficacy or neck lymph nodes treatment efficacy.

## DISCUSSION

Platinum-based chemotherapy concurrent with radiotherapy is widely used for the treatment of NPC, however, the therapeutic response and toxicity varies remarkably among individuals. Evidence of genetic polymorphisms underlying interindividual differences in chemoradiotherapy responses is rapidly increasing. For example, *XRCC1* rs25487 (A>G) was associated with increased risk of acute dermatitis and mucositis as well as poor progression free survival in NPC patients after curative chemoradiotherapy [22, 23]. However, others indicated rs25487 (A>G) was linked with decreased risk of severe fibrosis in NPC patients [24]. Results from Arab showed *MDM2* rs2279744 (T>G) appeared to have protective effect for late severe fibrosis and survival time among NPC patients [24]. Nevertheless, Liu *et al.* reported rs2279744 (T>G) as a risk factor for progression-free survival among southern Chinese NPC patients [25]. As far as we can see, most of the studies focused on protein coding genes lies in DNA damage repair pathway and the results were inconsistent in regard to different ethnicity, heterogeneous clinical confounding factors, limited sample size and so on. To the best of our knowledge, most of this research field is still blank, and the importance of lncRNA polymorphisms and chemoradiotherapy responses in NPC patients in Chinese remains unknown.

LncRNA *GAS5* is a rising star among tumor-suppressive lncRNAs. It is well known that effective control of both cell survival and cell proliferation is critical to successful cancer therapy. *GAS5* that accumulates in growth-arrested cells may alter cell fate by making the cells more or less susceptible to apoptotic and other growth-related stimuli through modulation of steroid hormone activities [18]. In *vitro* and in *vivo* results have shown that cell apoptosis induced by UV irradiation and chemotherapeutic drugs was augmented in cells over expression *GAS5*, and attenuated following down-regulation of *GAS5* expression [9, 11, 26, 27]. The apoptosis extent and the cell viability were quantitatively associated with the extent of *GAS5* level. In addition, *GAS5* expression was significantly downregulated in trastuzumab-resistant breast cancer patients, gefitinib-resistant lung adenocarcinoma cell lines and erlotinib-resistant glioma cell line, which was of great clinical significance for targeted therapy [28-30]. Emerging evidence have suggested *GAS5* as an indicator of overall survival in hepatocellular carcinoma, cervical cancer, gastric cancer, breast cancer, and colorectal cancer [15, 16, 21, 29, 31]. However, far less is known about the role of *GAS5* polymorphisms and chemoradiotherapy response in NPC patients.

In particular, we assessed correlations between three SNPs of *GAS5* and CCRT response and toxic reactions in 267 NPC patients, validating the findings in an additional 238 NPC patients from southern China. We found for the first time that GAS5 rs2067079 (C>T) exhibited a consistent association with CCRT induced severe myelosuppression and severe neutropenia in the discovery set (OR=2.403, P=0.009; OR=2.454, P=0.015; respectively), the validation set (OR=3.653, P=0.027; OR=4.767, P=0.016; respectively), and the combined dataset (OR=1.880, P=0.007; OR=2.079, P=0.005; respectively). Besides, we found an evidence of gene-dose effect for rs6790 on severe myelosuppression (GG *vs* GA *vs* AA: 23.56% *vs* 17.21% *vs* 10%) and severe neutropenia (GG *vs* GA *vs* AA: 30.4% *vs* 20.9% *vs* 17.1%), with the risk of toxic reactions decreased gradually along with the favorable allele increased. The statistical power for rs2067079 and rs6790 was 1.000 in the combined dataset.

**Figure 3:**
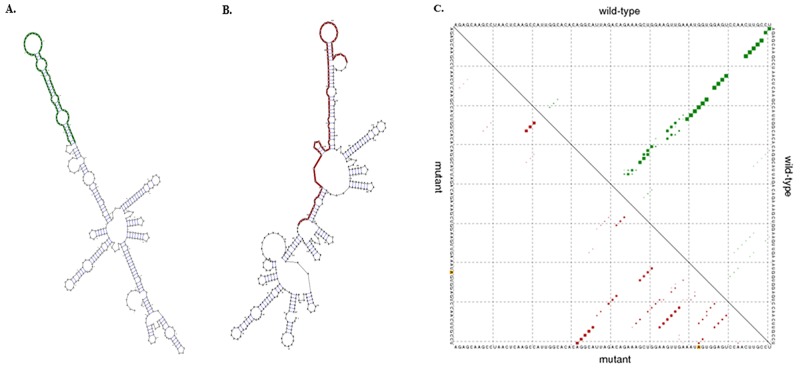
The predicted effect of rs2067079 on GAS5 secondary structure **(A)** Minimum free energy structures of the wild-type GAS5. **(B)** Minimum free energy structures of the mutant-type GAS5. **(C)** Dot plot of the GAS5 rs2067079 global structure. The upper and lower triangle of the matrix represents the base pair probabilities of wild-type and mutant sequences, respectively. The asymmetry of the two triangle matrix indicates the difference in their structure change.

Never had any study has reported about *GAS5* rs2067079 and rs6790, so we tried to infer the potential function of rs2067079 and rs6790 from some bioinformatics databases. The UCSC genome browser labeled the position of rs2067079 and rs6790 as an active promoter or strong enhancer region according to computationally integrating ChIP-seq data from nine human cell types. Since expression levels could be regulated by genetic variants in regulatory elements, we speculated that the two SNPs might influence *GAS5* transcriptional activity. In silico analysis revealed that rs2067079 and rs6790 both have a strong feature of expression Quantitative Trait Locus (e-QTL) in many tissues, indicating the two SNPs might exert their role through impacting the expression of target genes (Supplementary Table 4). Moreover, rs2067079 (C>T) had an obvious effect on *GAS5* secondary structure with the minimum free energy (MFE) increased from -111.50 kcal/mol to -109.10 kcal/mol (Figure 3). Considering that the spatial structure was critical for the performance of lncRNA function, we speculated rs2067079 might impact *GAS5* transcript stability, thus has a further implication. Both of TargetScan and miRanda predicted that rs2067079 caused three miRNA binding site gain (miR-4727-5p, miR-4769-3p, and miR-6817-5p) and one miRNA binding site loss (miR-6084) for *GAS5* (Supplementary Table 5), which inspired us to wonder if there existed a lncRNA-miRNA sponge role of *GAS5*. Yet, rs6790 didn’t have any effect on the interaction of miRNA and *GAS5*, indicating there might have other mechanisms for rs6790.

Though we found some positive associations in the present study, a number of critical points needed to be addressed in order to successfully unravel the genetics of chemoradiotherapy response in the future studies. It is noteworthy that the results of this study require to be validated in another population, since all involved patients were from the same hospital in Hunan province. A multicenter national study will further confirm the clinical value of these predictive biomarkers [32]. Given the fact that chemoradiotherapy response are complex phenotype and affected by multiple factors, it is very difficult to accurately predict their occurrence using a few SNPs. Microarray may help to map the tanglesome traits of the allelic architecture presumably underlying the complex phenotypes. Besides, establishment of risk model including genetic factors and clinical covariants capable of predicting individual likelihood of experiencing treatment resistance or developing side effects after chemoradiotherapy is of clinical significance, which may support the patient and the physician in selecting the best therapeutic approach and avoid unnecessary worsening of quality of life [33, 34].

In conclusion, in this two stage discovery-validation study, we highlighted two potential functional locus, *GAS5* rs2067079 and rs6790 for chemoradiotherapy induced severe myelosuppression and severe neutropenia among NPC patients for the first time. Our result provides valuable information for personalized chemoradiotherapy though there is still a long way to go.

## MATERIALS AND METHODS

### Study subjects recruitment

This study used a two stage discovery-validation approach. A total of 505 newly diagnosed histologically confirmed NPC patients were recruited in Hunan Provincial Cancer Hospital between October 2014 and July 2016. Among the whole patient cohort, 267 patients were assigned to discovery set (October 2014-September 2015), and 238 patients were assigned to validation set (October 2015-July 2016) according to their recruitment date. Patients were enrolled in this study according to the following inclusion criteria: (1) histological confirmed WHO type II or III NPC; (2) Han Chinese ethnicity; (3) received intensity-modulated radiotherapy (IMRT) technique; (4) treated with induction chemotherapy (IC) plus concurrent chemoradiotherapy (CCRT) regimen; (5) had complete follow-ups and clinical information; (6) had an Eastern Cooperative Oncology Group (ECOG) performance status of 0 or 1. The exclusion criteria including: (1) the presence of distant metastasis or recurrence; (2) underwent radiotherapy or chemotherapy or surgery before; (3) had a second malignancy or other concomitant malignant diseases.

This study was performed with the approval of the Independent Ethical Committee of Institute of Clinical Pharmacology, Central South University (CTXY-140007-2). At recruitment, written informed consent was obtained from all participants involved in this study.

### Treatment regimen

All the eligible patients received definitive radiotherapy and treated with platinum-based CCRT with IC regimen. The primary tumor and neck lymph nodes were treated with megavoltage photons (6 MV). Radiation was administered five times per week at a dose of 2.27 Gy/day. The accumulated radiation dose was 68-72 Gy to the primary tumor, 60-62 Gy to involved areas of the neck, and 50 Gy to uninvolved areas. There were six chemotherapy regimens involved in this study: platinum plus 5-fluorouracil (FP); platinum plus paclitaxel (TP); platinum plus docetaxel (DP); platinum plus gemcitabine (GP); cisplatin alone (DDP); nedaplatin alone (NDP). The selection of different regimens in individual patients was according to the patient’s clinical status, including performance status, symptoms, tumor burden, ongoing clinical trial and so on.

### Evaluation

We reviewed patients’ medical records during the follow-up period to collect demographic data and clinical information. The tumor, node, metastasis classification and tumor staging were evaluated according to the 2002 American Joint Committee on Cancer (AJCC) staging system. The toxic reactions were evaluated weekly during the CCRT treatment period by the Common Terminology Criteria for Adverse Events (CTCAE 3.0) for every patient. Grade 3-4 toxic reactions were considered as severe toxicity and Grade 1-2 were thought as mild toxicity. The response to chemoradiotherapy was evaluated using the Response Evaluation Criteria in Solid Tumor (RECIST) guidelines according to the magnetic resonance imaging (MRI) three months after treatment. A complete response (CR) and a partial response (PR) were considered as treatment sensitivity, whereas stable disease (SD) and progressive disease (PD) were identified as treatment resistance. The endpoints of this study were treatment efficacy, dermatitis, oral mucositis, myelosuppression, neutropenia, leukopenia, anemia, and thrombocytopenia.

### SNPs selection

Unlike the traditional tag SNP selection procedure, we used a bioinformatic approach to select SNPs with regulatory feature (5’-untranslated regions [5’- UTR] variant, 3’-untranslated regions [3’- UTR] variant, enhancer variant, splice region variant, miRNA binding site variant) by some online database, including ENCODE (http://genome.ucsc.edu/ENCODE/), ENSEMBL (http://asia.ensembl.org/index.html?redirect=no) and lncRNASNP (http://bioinfo.life.hust.edu.cn/lncRNASNP/). SNPs with a minor allele frequency (MAF) >5% in the Chinese population based on the 1000 Genomes CHS database were included for further study. Overall, 3 SNPs of GAS5 (rs2067079, rs6790, rs17359906) were finally identified, as shown in Table 2.

### DNA Extraction and genotyping

3 ml of venous blood was collected from each eligible participant at the time of enrollment and stored at -80°C. Genomic DNA was extracted from lymphocytes using the QIAamp DNA Blood Mini Kit (Qiagen, Valencia, CA) according to the manufacturer’s instructions. DNA purity and concentrations were determined by spectrophotometric measurement of absorbance at 260 and 280 nm by UV spectrophotometer. The candidate SNPs were genotyped using the Sequenom MassARRAY iPLEX platform (Sequenom, Inc., San Diego, CA, USA). A call-rate threshold of ≥95% was the criteria used to identify analyzable SNPs.

### Statistical analysis

The differences of demographic variables between two groups were evaluated by the Student’s t-test for continuous variables and the χ^2^ test for categorical variables. The Hardy-Weinberg equilibrium (HWE) was used to compare the observed genotype frequencies with the expected ones in discovery/validation/combined cohort. The associations between candidate SNPs and chemoradiotherapy efficacy and toxicities were estimated by calculating the adjusted odds ratios (OR) and their 95% confidence interval (CI) from multivariate logistic regression analyses, followed by stratification analysis. All tests were two-sided, and a P value <0.05 was considered the cutoff for statistical significance. The statistical analyses were performed with Statistical Package for Social Sciences software package (version 19.0 for Windows; Chicago, IL, USA) and R package.

## SUPPLEMENTARY MATERIALS FIGURE AND TABLES













## Abbreviations

NPC: nasopharyngeal carcinoma
SNPs: single nucleotide polymorphisms
lncRNA: Long non-coding RNA
GAS5: growth arrest-specific5
snoRNA: small nucleolar RNA
IMRT: intensity-modulated radiotherapy
IC: induction chemotherapy
CCRT: concurrent chemoradiotherapy
ECOG: Eastern Cooperative Oncology Group
FP: platinum plus 5-fluorouracil
TP: platinum plus paclitaxel
DP: platinum plus docetaxel
GP: platinum plus gemcitabine
DDP: cisplatin
NDP: nedaplatin
AJCC: American Joint Committee on Cancer
CTCAE: Common Terminology Criteria for Adverse Events
RECIST: Response Evaluation Criteria in Solid Tumor
MRI: magnetic resonance imaging
CR: complete response
PR: partial response
SD: stable disease
PD: progressive disease
MAF: minor allele frequency
HWE: Hardy-Weinberg equilibrium
OR: odds ratio
CI: confidence interval
ROC: receiver operating characteristic curve
AUC: area under the curve
e-QTL: expression Quantitative Trait Locus
MFE: minimum free energy
