# Supplementary material for: Genetic polymorphisms of long non-coding RNA GAS5 predict platinum-based concurrent chemoradiotherapy response in nasopharyngeal carcinoma patients
Source: Oncotarget. 2017 Jul 31;8(37):62286–97. doi: 10.18632/oncotarget.19725 (PMC5617505; doi:10.18632/oncotarget.19725)
Supplement: Supplementary file 1 [file oncotarget-08-62286-s001.pdf]

Genetic polymorphisms of long non-coding RNA GAS5 predict platinum-based concurrent chemoradiotherapy response in nasopharyngeal carcinoma patients

SUPPLEMENTARY MATERIALS

Supplementary Table 1: Multivariate logistic regression analysis of candidate SNPs and their association with concurrent chemoradiotherapy induced grade >2 leukopenia in NPC patients.

See Supplementry File 1

Supplementary Table 2: Multivariate logistic regression analysis of candidate SNPs and their association with concurrent chemoradiotherapy induced anemia in NPC patients.

See Supplementry File 2

Supplementary Table 3: Multivariate logistic regression analysis of candidate SNPs and their association with concurrent chemoradiotherapy induced grade >2 oral mucositis in NPC patients.

See Supplementry File 3

Supplementary Table 4: eQTL effect of rs2067079 and rs6790 in multi-tissues.

See Supplementry File 4

Supplementary Table 5: Prediction of candidate SNPs causing miRNA-lncRNA gain or loss by TargetScan and miRanda.

See Supplementry File 5
