# Supplementary material for: Genetic polymorphisms of long non-coding RNA GAS5 predict platinum-based concurrent chemoradiotherapy response in nasopharyngeal carcinoma patients
Source: Oncotarget. 2017 Jul 31;8(37):62286–97. doi: 10.18632/oncotarget.19725 (PMC5617505; doi:10.18632/oncotarget.19725)
Supplement: Supplementary file 2 [file oncotarget-08-62286-s002.docx]

**Supplementary Table 1. Multivariate logistic regression analysis of [candidate](D:/%E8%BD%AF%E4%BB%B6/Software/%E6%9C%89%E9%81%93%E8%AF%8D%E5%85%B8/Dict/6.3.69.8341/resultui/frame/javascript:void(0);) SNPs and their association with concurrent chemoradiotherapy induced grade >2 leukopenia in NPC patients**.

| Genotypes | Discovery Stage | | | | Validation Stage | | | | | Combined Stage | | | | |
| --- | --- | --- | --- | --- | --- | --- | --- | --- | --- | --- | --- | --- | --- | --- |
|  | Leukopenia | | OR^a^ (95% CI) | P ^a^ | Leukopenia | | | OR^a^ (95% CI) | P ^a^ | Leukopenia | | | OR^a^ (95% CI) | P ^a^ |
|  | Grade ≤2  N (%) | Grade >2  N (%) |  |  | Grade ≤2  N (%) | Grade >2  N (%) | |  |  | Grade ≤2  N (%) | Grade >2  N (%) | |  |  |
| rs2067079 |  |  |  |  |  | |  |  |  |  | |  |  |  |
| CC | 125(54.1) | 13(36.1) | 1.00 (reference) |  | 116 (57.7) | | 17 (45.9) | 1.00 (reference) |  | 241 (55.8) | | 30 (41.1) | 1.00 (reference) |  |
| CT | 81(35.1) | 23(63.9) | 2.938 (1.285-6.714) | **0.011** | 70 (34.8) | | 16 (43.2) | 1.362 (0.593-3.132) | 0.466 | 151 (35.0) | | 39 (53.4) | 1.901 (1.091-3.314) | **0.023** |
| TT | 25(10.8) | 0(0) | -- | -- | 13 (6.5) | | 4 (10.8) | 2.761 (0.666-11.455) | 0.162 | 38 (8.8) | | 4 (5.5) | 0.834 (0.260-2.672) | 0.759 |
| TT+CT vs CC |  |  | 2.326 (1.026-5.274) | **0.043** |  | |  | 1.532 (0.696-3.370) | 0.289 |  | |  | 1.700 (0.990-2.917) | 0.054 |
| TT vs CT+CC |  |  | -- | -- |  | |  | 2.418 (0.613-9.544) | 0.207 |  | |  | 0.626 (0.200-1.961) | 0.422 |
|  |  |  |  |  |  | |  |  |  |  | |  |  |  |
| rs6790 |  |  |  |  |  | |  |  |  |  | |  |  |  |
| GG | 97 (42.0) | 12 (33.3) | 1.00 (reference) |  | 65 (32.3) | | 17 (45.9) | 1.00 (reference) |  |  | |  | 1.00 (reference) |  |
| GA | 109 (47.2) | 22 (61.1) | 1.397 (0.597-3.269) | 0.441 | 98 (48.8) | | 15 (40.5) | 0.517 (0.229-1.170) | 0.114 | 162 (37.5) | | 29 (39.7) | 0.882 (0.498-1.561) | 0.666 |
| AA | 25 (10.8) | 2 (5.6) | 0.642 (0.120-3.422) | 0.604 | 38 (18.9) | | 5 (13.5) | 0.378 (0.120-1.195) | 0.098 | 207 (47.9) | | 37 (50.7) | 0.558 (0.219-1.417) | 0.220 |
| AA+GA vs GG |  |  | 1.250 (0.547-2.852) | 0.597 |  | |  | 0.476 (0.221-1.026) | 0.058 | 63 (14.6) | | 7 (9.6) | 0.807 (0.466-1.397) | 0.444 |
| AA vs GA+GG |  |  | 0.536 (0.108-2.662) | 0.446 |  | |  | 0.503 (0.167-1.516) | 0.222 |  | |  | 0.689 (0.375-1.266) | 0.230 |
|  |  |  |  |  |  | |  |  |  |  | |  |  |  |
| rs17359906 |  |  |  |  |  | |  |  |  |  | |  |  |  |
| GG | 209 (90.5) | 30 (83.3) | 1.00 (reference) |  | -- | | -- | -- |  | -- | | -- | -- |  |
| GA | 21 (9.1) | 6 (16.7) | 1.672 (0.522-5.355) | 0.387 | -- | | -- | -- | -- | -- | | -- | -- | -- |
| AA | 1 (0.4) | 0 (0) | -- | -- | -- | | -- | -- | -- | -- | | -- | -- | -- |
| AA+GA vs GG |  |  | 1.660 (0.519-5.316) | 0.393 |  | |  | -- | -- |  | |  | -- | -- |
| AA vs GA+GG |  |  | -- | -- |  | |  | -- | -- |  | |  | -- | -- |

^a^ Adjusted for gender, age, BMI, smoking status, drinking status, histological type, clinical stage, IC regimen, CCRT regimen, and pGTVnx irradiation dose.

P < 0.05 was shown in bold.
