# Supplementary material for: Genetic polymorphisms of long non-coding RNA GAS5 predict platinum-based concurrent chemoradiotherapy response in nasopharyngeal carcinoma patients
Source: Oncotarget. 2017 Jul 31;8(37):62286–97. doi: 10.18632/oncotarget.19725 (PMC5617505; doi:10.18632/oncotarget.19725)
Supplement: Supplementary file 3 [file oncotarget-08-62286-s003.docx]

**Supplementary Table 2. Multivariate logistic regression analysis of [candidate](D:/%E8%BD%AF%E4%BB%B6/Software/%E6%9C%89%E9%81%93%E8%AF%8D%E5%85%B8/Dict/6.3.69.8341/resultui/frame/javascript:void(0);) SNPs and their association with concurrent chemoradiotherapy induced anemia in NPC patients**.

| Genotypes | Discovery Stage | | | | Validation Stage | | | | Combined Stage | | | | |
| --- | --- | --- | --- | --- | --- | --- | --- | --- | --- | --- | --- | --- | --- |
|  | Anemia | | OR^a^ (95% CI) | P ^a^ | Anemia | | OR^a^ (95% CI) | P ^a^ | Anemia | | | OR^a^ (95% CI) | P ^a^ |
|  | Grade 0  N (%) | Grade >0  N (%) |  |  | Grade 0  N (%) | Grade >0  N (%) |  |  | Grade 0  N (%) | Grade >0  N (%) | |  |  |
| rs2067079 |  |  |  |  |  |  |  |  |  |  | |  |  |
| CC | 86 (53.1) | 52 (49.5) | 1.00 (reference) |  | 68 (54.0) | 65 (58.0) | 1.00 (reference) |  | 154 (53.5) | 117 (53.9) | | 1.00 (reference) |  |
| CT | 61 (37.7) | 43 (41.0) | 1.302 (0.676-2.510) | 0.430 | 49 (38.9) | 37 (33.0) | 0.632 (0.338-1.181) | 0.151 | 110 (38.2) | 80 (36.9) | | 0.861 (0.560-1.324) | 0.496 |
| TT | 15 (9.3) | 10 (9.5) | 1.432 (0.491-4.172) | 0.511 | 9 (7.1) | 8 (7.1) | 1.265 (0.397-4.027) | 0.691 | 24 (8.3) | 18 (8.3) | | 1.186 (0.561-2.506) | 0.655 |
| TT+CT vs CC |  |  | 1.327 (0.716-2.462) | 0.369 |  |  | 0.710 (0.394-1.279) | 0.255 |  |  | | 0.912 (0.608-1.370) | 0.658 |
| TT vs CT+CC |  |  | 1.277 (0.456-3.571) | 0.642 |  |  | 1.495 (0.484-4.618) | 0.485 |  |  | | 1.262 (0.610-2.608) | 0.530 |
|  |  |  |  |  |  |  |  |  |  | |  |  |  |
| rs6790 |  |  |  |  |  |  |  |  |  | |  |  |  |
| GG | 63(38.9) | 46(43.8) | 1.00 (reference) |  | 41 (32.5) | 41 (36.6) | 1.00 (reference) |  | 104 (36.1) | | 87 (40.1) | 1.00 (reference) |  |
| GA | 85(52.5) | 46(43.8) | 0.501 (0.258-0.937) | **0.041** | 61 (48.4) | 52 (46.4) | 0.823 (0.433-1.565) | 0.553 | 146 (50.7) | | 98 (45.2) | 0.712 (0.461-1.100) | 0.126 |
| AA | 14(8.6) | 13(12.4) | 0.921 (0.321-2.638) | 0.878 | 24 (19.0) | 19 (17.0) | 0.887 (0.386-2.042) | 0.779 | 38 (13.2) | | 32 (14.7) | 0.898 (0.478-1.687) | 0.738 |
| AA+GA vs GG |  |  | 0.564 (0.301-1.055) | 0.073 |  |  | 0.840 (0.459-1.539) | 0.573 |  | |  | 0.750 (0.496-1.132) | 0.171 |
| AA vs GA+GG |  |  | 1.309 (0.485-3.534) | 0.595 |  |  | 0.996 (0.474-2.090) | 0.991 |  | |  | 1.085 (0.606-1.942) | 0.783 |
|  |  |  |  |  |  |  |  |  |  | |  |  |  |
| rs17359906 |  |  |  |  |  |  |  |  |  | |  |  |  |
| GG | 146 (90.1) | 93 (88.6) | 1.00 (reference) |  | -- | -- | -- |  | -- | | -- | -- |  |
| GA | 15 (9.3) | 12 (11.4) | 1.400 (0.492-3.985) | 0.528 | -- | -- | -- | -- | -- | | -- | -- | -- |
| AA | 1 (0.6) | 0 (0) | -- | -- | -- | -- | -- | -- | -- | | -- | -- | -- |
| AA+GA vs GG |  |  | 1.380 (0.487-3.914) | 0.544 |  |  | -- | -- |  | |  | -- | -- |
| AA vs GA+GG |  |  | -- | -- |  |  | -- | -- |  | |  | -- | -- |

^a^ Adjusted for gender, age, BMI, smoking status, drinking status, histological type, clinical stage, IC regimen, CCRT regimen, and pGTVnx irradiation dose.

P < 0.05 was shown in bold.
