# Supplementary material for: Genetic polymorphisms of long non-coding RNA GAS5 predict platinum-based concurrent chemoradiotherapy response in nasopharyngeal carcinoma patients
Source: Oncotarget. 2017 Jul 31;8(37):62286–97. doi: 10.18632/oncotarget.19725 (PMC5617505; doi:10.18632/oncotarget.19725)
Supplement: Supplementary file 4 [file oncotarget-08-62286-s004.docx]

**Supplementary Table 3. Multivariate logistic regression analysis of [candidate](D:/%E8%BD%AF%E4%BB%B6/Software/%E6%9C%89%E9%81%93%E8%AF%8D%E5%85%B8/Dict/6.3.69.8341/resultui/frame/javascript:void(0);) SNPs and their association with concurrent chemoradiotherapy induced grade >2 oral mucositis in NPC patients**.

| Genotypes | Discovery Stage | | | | Validation Stage | | | | Combined Stage | | | | |
| --- | --- | --- | --- | --- | --- | --- | --- | --- | --- | --- | --- | --- | --- |
|  | Oral mucositis | | OR^a^ (95% CI) | P ^a^ | Oral mucositis | | OR^a^ (95% CI) | P ^a^ | Oral mucositis | | | OR^a^ (95% CI) | P ^a^ |
|  | Grade ≤2  N (%) | Grade >2  N (%) |  |  | Grade ≤2  N (%) | Grade >2  N (%) |  |  | Grade ≤2  N (%) | Grade >2  N (%) | |  |  |
| rs2067079 |  |  |  |  |  |  |  |  |  | |  |  |  |
| CC | 116(55.2) | 22(38.6) | 1.00 (reference) |  | 90 (54.2) | 43 (59.7) | 1.00 (reference) |  | 206 (54.8) | | 65 (50.4) | 1.00 (reference) |  |
| CT | 75(35.7) | 29(50.9) | 1.928 (1.004-3.701) | **0.049** | 62 (37.3) | 24 (33.3) | 0.723 (0.384-1.361) | 0.315 | 137 (36.4) | | 53 (41.1) | 1.177 (0.756-1.833) | 0.470 |
| TT | 19(9.1) | 6(10.5) | 1.532 (0.526-4.462) | 0.434 | 12 (7.2) | 5 (6.9) | 0.831 (0.257-2.683) | 0.757 | 31 (8.2) | | 11 (8.5) | 1.129 (0.523-2.439) | 0.757 |
| TT+CT vs CC |  |  | 1.846 (0.990-3.442) | 0.054 |  |  | 0.740 (0.408-1.344) | 0.323 |  | |  | 1.168 (0.768-1.777) | 0.467 |
| TT vs CT+CC |  |  | 1.082 (0.379-3.090) | 0.883 |  |  | 0.935 (0.296-2.950) | 0.908 |  | |  | 1.054 (0.500-2.222) | 0.891 |
|  |  |  |  |  |  |  |  |  |  | |  |  |  |
| rs6790 |  |  |  |  |  |  |  |  |  | |  |  |  |
| GG | 79(37.6) | 30(52.6) | 1.00 (reference) |  | 63 (38.0) | 19 (26.4) | 1.00 (reference) |  | 142 (37.8) | | 49 (38.0) | 1.00 (reference) |  |
| GA | 107(51.0) | 24(42.1) | 0.511 (0.262-0.998) | **0.049** | 74 (44.6) | 39 (54.2) | 1.764 (0.898-3.466) | 0.1 | 181 (48.1) | | 63 (48.8) | 0.960 (0.613-1.502) | 0.857 |
| AA | 24(11.4) | 3(5.3) | 0.352 (0.091-1.362) | 0.131 | 29 (17.5) | 14 (19.4) | 1.618 (0.684-3.827) | 0.274 | 53 (14.1) | | 17 (13.2) | 0.915 (0.474-1.769) | 0.792 |
| AA+GA vs GG |  |  | 0.486 (0.254-0.928) | **0.029** |  |  | 1.706 (0.926-3.142) | 0.086 |  | |  | 0.950 (0.620-1.456) | 0.814 |
| AA vs GA+GG |  |  | 0.501 (0.136-1.852) | 0.300 |  |  | 1.135 (0.541-2.381) | 0.737 |  | |  | 0.937 (0.511-1.720) | 0.834 |
|  |  |  |  |  |  |  |  |  |  | |  |  |  |
| rs17359906 |  |  |  |  |  |  |  |  |  | |  |  |  |
| GG | 189 (90.0) | 50 (87.7) | 1.00 (reference) |  | -- | -- | -- |  | -- | | -- | -- |  |
| GA | 21 (10.0) | 6 (1.5) | 0.900 (0.312-2.592) | 0.845 | -- | -- | -- | -- | -- | | -- | -- | -- |
| AA | 0 (0) | 1 (1.8) | -- | -- | -- | -- | -- | -- | -- | | -- | -- | -- |
| AA+GA vs GG |  |  | 1.036 (0.378-2.839) | 0.945 |  |  | -- | -- |  | |  | -- | -- |
| AA vs GA+GG |  |  | -- | -- |  |  | -- | -- |  | |  | -- | -- |

^a^ Adjusted for gender, age, BMI, smoking status, drinking status, histological type, clinical stage, IC regimen, CCRT regimen, and pGTVnx irradiation dose.

P < 0.05 was shown in bold.
