# Supplementary material for: Genetic polymorphisms of long non-coding RNA GAS5 predict platinum-based concurrent chemoradiotherapy response in nasopharyngeal carcinoma patients
Source: Oncotarget. 2017 Jul 31;8(37):62286–97. doi: 10.18632/oncotarget.19725 (PMC5617505; doi:10.18632/oncotarget.19725)
Supplement: Supplementary file 5 [file oncotarget-08-62286-s005.docx]

**Supplementary Table 4. eQTL effect of rs2067079 and rs6790 in multi-tissues.**

| Gencode Id | Gene | P value | Effect size | Tissue |
| --- | --- | --- | --- | --- |
| eQTLs of rs2067079 in multi-tissue | | | | |
| ENSG00000117593.8 | [DARS2](http://www.gtexportal.org/home/snp/javascript:portalClient.eqtl.gotoGeneExpression('DARS2')) | 4.3e-17 | -0.40 | Muscle - Skeletal |
| ENSG00000183831.6 | [ANKRD45](http://www.gtexportal.org/home/snp/javascript:portalClient.eqtl.gotoGeneExpression('ANKRD45')) | 4.5e-13 | -0.55 | Testis |
| ENSG00000117601.9 | [SERPINC1](http://www.gtexportal.org/home/snp/javascript:portalClient.eqtl.gotoGeneExpression('SERPINC1')) | 4.4e-8 | 0.57 | Esophagus - Muscularis |
| ENSG00000152061.17 | RABGAP1L | 7.0e-7 | 0.35 | Breast - Mammary Tissue |
| ENSG00000152061.17 | RABGAP1L | 0.02 | 0.107 | Whole Blood |
| ENSG00000135870.7 | RC3H1 | 0.05 | -0.048 | Whole Blood |
| eQTLs of rs6790 in multi-tissue | | | | |
| ENSG00000270084.1 | GAS5-AS1 | 2.6e-8 | -1.0 | Colon - Transverse |
| ENSG00000152061.17 | RABGAP1L | 7.2e-7 | 0.43 | Adipose - Subcutaneous |
| ENSG00000183831.6 | ANKRD45 | 0.0000042 | 0.56 | Cells - Transformed fibroblasts |
| ENSG00000152061.17 | RABGAP1L | 0.0000095 | 0.32 | Skin - Sun Exposed (Lower leg) |
| ENSG00000270084.1 | GAS5-AS1 | 0.000014 | -0.38 | Cells - Transformed fibroblasts |
| ENSG00000270084.1 | GAS5-AS1 | 0.000055 | -0.84 | Adrenal Gland |
| ENSG00000152061.17 | RABGAP1L | 0.000065 | 0.37 | Cells - Transformed fibroblasts |

Data Source: GTEx Analysis Release V6p (dbGaP Accession phs000424.v6.p1)
