# Supplementary material for: Genetic polymorphisms of long non-coding RNA GAS5 predict platinum-based concurrent chemoradiotherapy response in nasopharyngeal carcinoma patients
Source: Oncotarget. 2017 Jul 31;8(37):62286–97. doi: 10.18632/oncotarget.19725 (PMC5617505; doi:10.18632/oncotarget.19725)
Supplement: Supplementary file 6 [file oncotarget-08-62286-s006.docx]

**Supplementary Table 5. Prediction of candidate SNPs causing miRNA-lncRNA gain or loss by TargetScan and miRanda.**

| SNP | SNP causes miRNA-lncRNA gain | SNP causes miRNA-lncRNA loss |
| --- | --- | --- |
| rs2067079 | [hsa-miR-4727-5p](http://bioinfo.life.hust.edu.cn/lncRNASNP/mirnas/show?id=hsa-miR-4727-5p)  miRNA:  3' ggugacaccuucgACCGUCUa 5'                         \|\|\|\|**\|**\|\| lncRNA: 5' tgcattaaataaaTGGCAGAt 3' | [hsa-miR-6084](http://bioinfo.life.hust.edu.cn/lncRNASNP/mirnas/show?id=hsa-miR-6084)  miRNA:  3' ggccgguggcUGACCGCCUu 5'                      \| \|\|\|\|**X**\|\| lncRNA: 5' gcattaaataAATGGCGGAt 3' |
|  | hsa-miR-4769-3p  miRNA:  3' cauccccucccuccUACCGUCu 5'                          \|\|\|\|\|**\|**\| lncRNA: 5' aatgcattaaataaATGGCAGa 3' |  |
|  | hsa-miR-6817-5p  miRNA:  3' ggugagguucgaaggaUACCGUCu 5'                            \|\|\|\|\|**\|**\| lncRNA: 5' tgaatgcattaaataaATGGCAGa 3' |  |
| rs6790 | no | no |
| rs17359906 | hsa-miR-1297  miRNA:  3' guggacuuaAUGAACUu 5'                     \|\|\|**\|**\|\|\| lncRNA: 5' taaaatctgTACTTGAt 3' | hsa-miR-4772-5p  miRNA:  3' ucagacgUUA-AAACGGACUAGu 5'                   \|\|\| \| \| \|**X**\|\|\|\|\| lncRNA: 5' gaattaaAATCTGTACCTGATCt 3' |
|  | hsa-miR-26a-5p  miRNA:  3' ucggaUAGGACCUAAUGAACUu 5'                 \| :\|\|\|   \|\|\|**\|**\|\|\| lncRNA: 5' attaaAATCTG---TACTTGAt 3' |  |
|  | hsa-miR-26b-5p  miRNA:  3' uggaUAGGACUUAAUGAACUu 5'                \| :\|\|\|   \|\|\|**\|**\|\|\| lncRNA: 5' ttaaAATCTG---TACTTGAt 3' |  |
